# A Spectrum of Neural Autoantigens, Newly Identified by Histo-Immunoprecipitation, Mass Spectrometry, and Recombinant Cell-Based Indirect Immunofluorescence

**DOI:** 10.3389/fimmu.2018.01447

**Published:** 2018-07-09

**Authors:** Madeleine Scharf, Ramona Miske, Stephanie Kade, Stefanie Hahn, Yvonne Denno, Nora Begemann, Nadine Rochow, Christiane Radzimski, Stephanie Brakopp, Christian Probst, Bianca Teegen, Winfried Stöcker, Lars Komorowski

**Affiliations:** Institute of Experimental Immunology, EUROIMMUN AG, Lübeck, Germany

**Keywords:** neural autoantibodies, immunoprecipitation, antigen identification, autoantigens, indirect immunofluorescence

## Abstract

**Background:**

A plurality of neurological syndromes is associated with autoantibodies against neural antigens relevant for diagnosis and therapy. Identification of these antigens is crucial to understand the pathogenesis and to develop specific immunoassays. Using an indirect immunofluorescence assay (IFA)-based approach and applying different immunoprecipitation (IP), chromatographic and mass spectrometric protocols was possible to isolate and identify a spectrum of autoantigens from brain tissue.

**Methods:**

Sera and CSF of 320 patients suspected of suffering from an autoimmune neurological syndrome were comprehensively investigated for the presence of anti-neural IgG autoantibodies by IFA using mosaics of biochips with brain tissue cryosections and established cell-based recombinant antigen substrates as well as immunoblots. Samples containing unknown brain tissue-specific autoantibodies were subjected to IP with cryosections of cerebellum and hippocampus (rat, pig, and monkey) immobilized to glass slides or with lysates produced from homogenized tissue, followed by sodium dodecyl sulfate-polyacrylamide gel electrophoresis, tryptic digestion, and matrix-assisted laser desorption/ionization–time of flight mass spectrometry analysis. Identifications were confirmed by IFA with recombinant HEK293 cells and by neutralizing the patients’ autoantibodies with the respective recombinantly expressed antigens in the tissue-based immunofluorescence test.

**Results:**

Most samples used in this study produced speckled, granular, or homogenous stainings of the hippocampal and cerebellar molecular and/or granular layers. Others exclusively stained the Purkinje cells. Up to now, more than 20 different autoantigens could be identified by this approach, among them ATP1A3, CPT1C, Flotillin1/2, ITPR1, NBCe1, NCDN, RGS8, ROCK2, and Syntaxin-1B as novel autoantigens.

**Discussion:**

The presented antigen identification strategy offers an opportunity for identifying up to now unknown neural autoantigens. Recombinant cell substrates containing the newly identified antigens can be used in serology and the clinical relevance of the autoantibodies can be rapidly evaluated in cohort studies.

## Introduction

In autoimmune diseases, an abnormal response of the immune system attacks the body’s own cells causing malfunction or injury. The immune response is often associated with the appearance of autoreactive antibodies (=autoantibodies) that bind specifically to the body’s own structures (=autoantigens). In neurological autoimmune diseases the nervous system is affected as a result. The spectrum of diagnoses of neurological autoimmune disorders has expanded rapidly in the recent years due to the discovery of new anti-neural antibodies.

Most of the initially described anti-neural autoantibodies are directed against intracellular proteins like Hu, Yo, Ri, Ta, GAD, and amphiphysin (1). They are generally considered to be epiphenomena of a T-cell-driven reaction against tumor cells expressing neuronal antigens. Because of their limited access to their target antigens, they probably bear no pathogenic potential *in vivo* though anti-Hu has recently been shown to activate neurons (2). They are crucial biomarkers for the diagnosis of paraneoplastic autoimmune disorders and often lead to very early diagnosis of the corresponding cancers.

In the past years, a significant number of pathogenic autoantibodies against neural surface-associated proteins have been described, including AQP4, NMDAR, LGI1, CASPR2, AMPAR1, and AMPAR2, GABA-A and -B receptors, glycin receptor, DPPX, mGluR5, IgLON5, and neurexin-3-alpha (1, 3–7). These autoantibodies are frequently non-paraneoplastic, generally occur in association with inflammatory damage to the brain and can trigger seizures, impairment of visual acuity, psychosis-like symptoms, and/or movement disorders.

Most notably, anti-neural autoantibodies are not only important diagnostic markers. Knowing the identity of the corresponding autoantigens also helps to determine the treatment strategy. Patients with antibodies targeting intracellular proteins generally respond poorly to immunosuppressive treatments but need fast onset of oncotherapy in most cases. In contrast, antibodies targeting cell surface proteins can have direct pathogenic effects, and patients’ symptoms often improve after immunotherapy.

The most frequently used method for neural cell surface antigen identification has been immunoprecipitation after antibody binding to live primary hippocampal neurons (5–11). The neuronal cells used for these experiments are isolated from rat embryos and need to be cultivated for at least 14 days to allow differentiation before patient antibodies are applied to the culture. Preparation and cultivation of the cells are elaborate, thus hampering their use for screening of autoantibodies in clinical diagnostics. Moreover, the method is limited as it can only be used if the antigenic target is presented at the surface of the cultivated neurons.

For identification of intracellular antigens, immunoscreening of cDNA expression libraries (12, 13) or libraries of purified recombinant proteins (14) has been used successfully. Other strategies rely on the separation of brain tissue extracts in one- or two-dimensional gel electrophoresis followed by transfer of the separated proteins onto membranes and incubation with patient antibodies (15, 16). In these experiments, the frequency of irrelevant positive results is high because revelation of hidden epitopes may lead to unspecific binding of antibodies to unfolded and/or electrophoretically concentrated proteins. At the same time, these methods often denature the three-dimensional structure of protein antigens. Since autoantibodies sometimes do not bind to structurally non-authentic antigens in these setups, their usefulness is further limited.

Here, we report on an antigen identification strategy that is derived from indirect immunofluorescence assay (IFA) using brain tissue cryosections which is still one of the most versatile screening procedures for anti-neural autoantibodies, especially against up to now unspecified target antigens. Cryosectioning generally conserves the microenvironment of tissues such that protein structures are stabilized and epitopes presented nearly authentical.

## Materials and Methods

In the first step, the immunocomplexes formed by the patient’s autoantibodies and the animal tissue cryosections were analyzed in an immunocomplex extraction assay. A combination of histo- or tissue-immunoprecipitation (HIP/TIP), chromatography, and mass spectrometry was used for antigen identification. Correct antigen identification was verified by IFA using the respective antigen recombinantly expressed in HEK293 cells and by neutralizing the autoantibodies’ tissue reaction with the recombinant antigen.

### Reagents

Reagents were obtained from Merck, Darmstadt, Germany, or Sigma-Aldrich, Heidelberg, Germany if not specified otherwise.

### Human Samples

An ethics approval was not required as per institutional and national guidelines. The serum samples were collected by the clinical immunological laboratory Stöcker, Lübeck (Germany) for the purpose of autoantibody testing and were provided to the authors in an anonymized form. Hence, the authors did not have access to identifiable information. After use for clinical diagnostics the samples were coded and stored at −20°C until first research use after which they were stored at +4°C for further experiments to avoid repeated freeze/thaw cycles. Stored sera samples were used as sources of IgG antibodies.

Control collectives included healthy blood donors and patients with defined autoantibody-associated neurological syndromes.

### Indirect Immunofluorescence Assay

Indirect immunofluorescence assay was conducted using slides with a diagnostic biochip screening array of brain tissue cryosections (hippocampus of rat, cerebellum of rat and monkey) combined with recombinant HEK293 cells, each biochip separately expressing the following 30 brain antigens: Hu, Yo, Ri, CV2, SOX1, PNMA1, PNMA2, ARHGAP26, Homer3, CARP VIII, ZIC4, DNER/Tr, GAD65, GAD67, amphiphysin, recoverin, GABAB receptor, glycine receptor, DPPX, glutamate receptors (types NMDA, AMPA, mGluR1, mGluR5), LGI1, CASPR2, AQP4 (M1 and M23), MOG, MP-0, and MAG. Each biochip mosaic was incubated with 70 µL of PBS-diluted sample at room temperature for 30 min, washed with PBS-Tween for 5 min. In the second step, either Alexa488-labeled goat anti-human IgG (Jackson Research, Suffolk, United Kingdom), or fluorescein isothiocyanate-labeled goat anti-human IgG (Euroimmun) were applied and incubated at room temperature for 30 min. Slides were washed again with PBS-Tween for 5 min. Slides were embedded in PBS-buffered, DABCO containing glycerol, and examined by fluorescence microscopy. Positive and negative controls were included. Samples were classified as positive or negative based on fluorescence reactivity of the transfected cells in direct comparison with mock-transfected cells and control samples. Cell nuclei were visualized by DNA staining with TO-PRO3 iodide (dilution 1:2,000) (ThermoFisher Scientific, Schwerte, Germany). In neutralization experiments, recombinant antigens were mixed with diluted serum samples 1 h prior to the IFA as described in Stöcker et al. (17). Results were evaluated independently by two observers using a laser scanning microscope (LSM700, Zeiss, Jena, Germany).

### Immunocomplex Extraction Assay

The assay was performed using slides with a biochip array of brain tissue cryosections (rat hippocampus, rat cerebellum, and monkey cerebellum). Each biochip mosaic was incubated with PBS-diluted sample at room temperature for 30 min, washed with PBS-Tween, and immersed in PBS-Tween for 5 min. In the second step, the slides were incubated either in extraction buffer [100 mmol/L Tris–HCl pH 7.4, 150 mmol/L sodium chloride, 2.5 mmol/L ethylenediamine tetraacetic acid (EDTA), 0.5% (w/v) deoxycholate, and 1% (w/v) Triton X-100 containing protease inhibitors] or in detergent-free control buffer for 30 min at room temperature while gently shaking. After washing with PBS-Tween, the incubation with secondary antibody, washing, and preparation of slides fluorescence microscopy was performed as described for IFA. Extraction of the immunocomplexes was estimated by direct comparison of the signal intensity of the biochip mosaics incubated with extraction buffer or with the control buffer.

### Histo-Immunoprecipitation

Cerebellum from rat or pig was dissected and shock-frozen in −160°C isopentane. The tissue was cryosectioned (4 µm) with a SM2000R microtome (Leica Microsystems, Nussloch, Germany), placed on the entire surface of glass slides, and dried. Whole slides were then incubated with patient’s serum (diluted 1:100) at 4°C for 3 h followed by three washing steps with PBS containing 0.2% (w/v) Tween 20. Immunocomplexes were extracted from the sections by incubation in extraction buffer [100 mmol/L Tris–HCl pH 7.4, 150 mmol/L sodium chloride, 2.5 mmol/L EDTA, 0.5% (w/v) deoxycholate, and 1% (w/v) Triton X-100 containing protease inhibitors] at room temperature for 30 min. Detached material was homogenized and centrifuged at 16,000 × *g* at 4°C for 15 min. The clear supernatants were then incubated with Protein G Dynabeads (ThermoFisher Scientific, Dreieich, Germany) at 4°C overnight to capture immunocomplexes. The beads were washed three times with PBS and eluted with NuPage LDS sample buffer (ThermoFisher Scientific, Schwerte, Germany) containing 25 mmol/L dithiothreitol at 70°C for 10 min. Carbamidomethylation with 59 mM iodoacetamide (Bio-Rad, Hamburg, Germany) was performed prior to sodium dodecyl sulfate (SDS)-polyacrylamide gel electrophoresis (PAGE) (NuPAGE, ThermoFisher Scientific, Schwerte, Germany). Separated proteins were visualized with Coomassie Brilliant Blue (G-250) (Merck) and identified by mass spectrometric (MS) analysis (see below for details).

### Tissue-Immunoprecipitation

Hippocampus or cerebellum from rat was dissected and shock-frozen in liquid nitrogen. The tissues were homogenized in extraction buffer [100 mmol/L Tris–HCl pH 7.4, 150 mmol/L sodium chloride, 2.5 mmol/L EDTA, 0.5% (w/v) sodium deoxycholate, and 1% (w/v) Triton X-100] containing protease inhibitors (Complete mini, Roche Diagnostics, Penzberg, Germany) with a Miccra D-8 (Roth, Karlsruhe, Germany) and a hand homogenizer (Sartorius, Göttingen, Germany) at 4°C. The tissue lysates was centrifuged at 21,000 × *g* at 4°C for 15 min and clear supernatants were incubated with patient’s serum (diluted 1:33) at 4°C for 3 h. Pulldown of immunocomplexes and analysis of eluate fractions was performed as described for HIP.

### Mass Spectrometry

Mass spectrometry sample preparation was performed similar to Koy et al. (18). Unless otherwise indicated, hardware, software, MALDI targets, peptide standards, and matrix reagents were obtained from Bruker Daltonics, Bremen, Germany.

Briefly, visible protein bands were excised from Coomassie Brilliant Blue G-250 stained gels. After destaining and tryptic digestion peptides were extracted and spotted with α-cyano-4-hydroxycinnamic acid onto a MTP AnchorChip™ 384 TF target.

Matrix-assisted laser desorption/ionization–time of flight mass spectrometry/TOF measurements were performed with an Autoflex III smartbeam TOF/TOF200 System using flexControl 3.0, 3.3, or 3.4 software. MS spectra for peptide mass fingerprinting (PMF) were recorded in positive ion reflector mode with 4,000–10,000 shots and in a mass range from 600 to 4,000 Da. Spectra were calibrated externally with the commercially available Peptide Calibration Standard II, processed with flexAnalysis 3.0, 3.3, or 3.4 and peak lists were analyzed with BioTools 3.2.

The Mascot search engine Mascot Server 2.3 (Matrix Science, London, UK) was used for protein identification by searching against the NCBI or SwissProt database limited to Mammalia. Search parameters were as follows: mass tolerance was set to 80 ppm, one missed cleavage site was accepted, and carbamidomethylation of cysteine residues as well as oxidation of methionine residues were set as fixed and variable modifications, respectively. To evaluate the protein hits, a significance threshold of *p* < 0.05 was chosen.

For further confirmation of the PMF hits two to five peptides of each identified protein were selected for MS/MS measurements using the WARP feedback mechanism of BioTools. Parent and fragment masses were recorded with 400 and 1,000 shots, respectively. Spectra were processed and analyzed as described above with a fragment mass tolerance of 0.7 Da.

### Recombinant Expression of Antigens in HEK293 Cells

For cloning details see Table S1 in Supplementary Material. The antigens were expressed in the human cell line HEK293 after ExGen500-mediated transfection (ThermoFisher Scientific) according to the manufacturer’s instructions.

For the preparation of IFA substrates, HEK293 were grown on sterile cover glasses, transfected, and allowed to express the recombinant antigens for 48 h. Cover glasses were washed with PBS, fixed with acetone for 10 min at room temperature, air-dried, cut into 2 mm × 2 mm-sized fragments (biochips) and used as substrates in IFA as described. Alternatively, cells were transfected in standard T-flasks and the cells harvested after 48 h. The cell suspension was centrifuged at 1,500 × *g*, 4°C for 20 min and the resulting sediment extracted with 20 mmol/L Tris–HCl pH 7.4, 50 mmol/L potassium chloride, 5 mmol/L EDTA. The extracts were stored in aliquots at −80°C until further use.

## Results

### Results of Serum Prescreening by IFA

The samples used in this study had originally been sent to the Clinical Immunological Laboratory for determination of anti-neural autoantibodies between 01/2011 and 07/2016. The 320 sera included in this study showed different IgG staining patterns on brain tissue cryosections (hippocampus of rat, cerebellum of rat and monkey) with a minimum endpoint titer of 1:100 (Figure S1 in Supplementary Material). Most samples (*n* = 261) produced speckled, granular, or homogenous staining of the hippocampal and cerebellar molecular and granular layers. Others (*n* = 59) stained cerebellar Purkinje cells. None of these sera revealed reactivity with the monospecific biochips of the diagnostic biochip screening array.

### Immunocomplex Extractability

**Figure 1:**
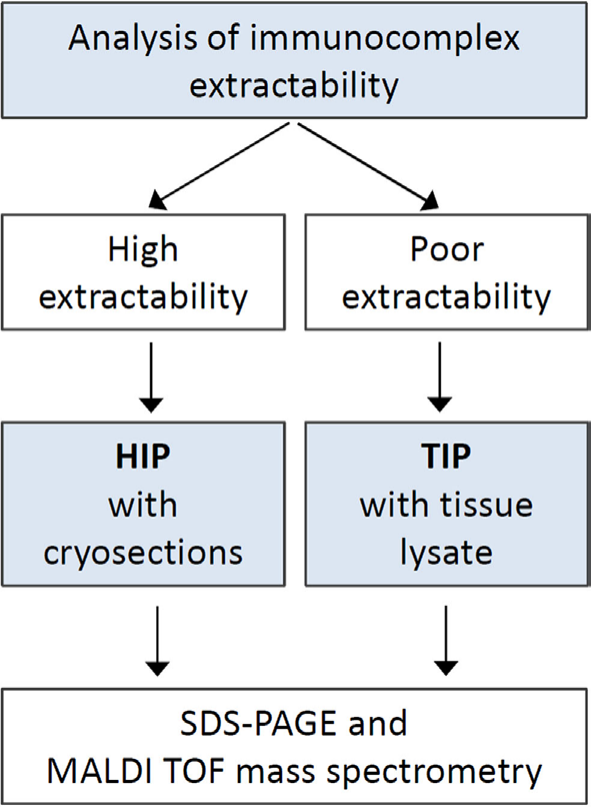
Procedure diagram. Schematic representation of the steps of antigen identification. Abbreviations: HIP, histo-immunoprecipitation; TIP, tissue-immunoprecipitation.

The extractability of immunocomplexes from cryosections bound to the glass slide surface was determined for each serum sample prior to immunoprecipitation. For sera which showed medium to high extractability (*n* = 182), HIP was applied, while for the other sera TIP was used (*n* = 138) (Figure 1).

**Figure 2:**
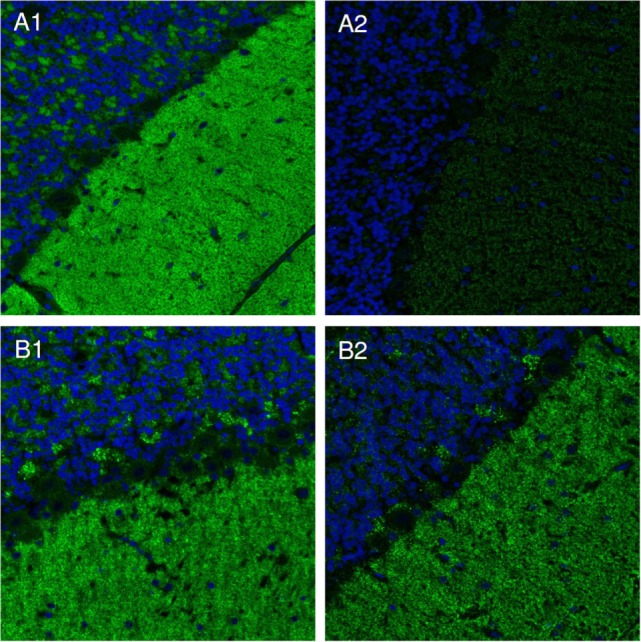
Immunocomplex extraction. Slides with cerebellum cryosections were incubated with patients’ sera and subsequently with either extraction [100 mmol/L Tris–HCl pH 7.4, 150 mmol/L sodium chloride, 2.5 mmol/L ethylenediamine tetraacetic acid, 0.5% (w/v) deoxycholate, 1% (w/v) Triton X-100] **(A2,B2)**, or detergent-free control buffer **(A1,B1)**. Bound IgG was visualized by Alexa488-labeled goat anti-human IgG. Nuclei were counterstained by incubation with TO-PRO-3 iodide (blue). Examples of soluble **(A)** and insoluble **(B)** immunocomplexes are shown.

Some sera did not show any differences in the signal intensities after using extraction and control buffer, while other sera showed slight or even strong reduction of signal intensity after incubation with extraction buffer (Figure 2). A strong decrease of signal intensity was interpreted as high extractability of the respective immunocomplexes.

### Identified Autoantigens

**Figure 3:**
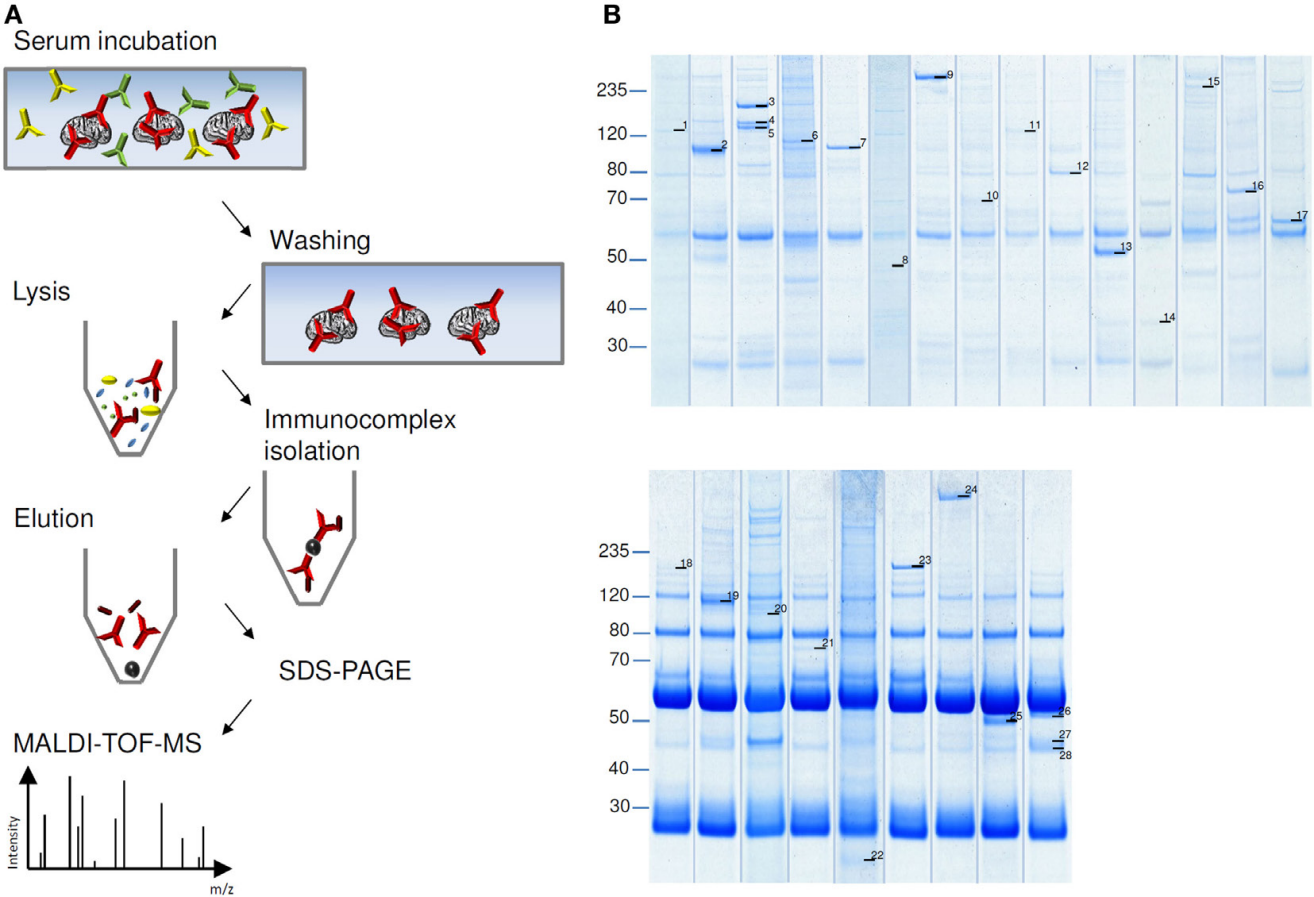
Immunoprecipitation of autoantigens. **(A)** Schema of histo-immunoprecipitation (HIP) with cerebellum cryosections immobilized on glass slides. Slides were incubated with patient’s serum followed by washing and removal of unbound antibodies. Cells were lysed and soluble immunocomplexes were isolated by protein-G-coated magnetic beads. Sodium dodecyl sulfate (SDS)-eluted proteins were separated by SDS-polyacrylamide gel electrophoresis and visualized by staining with colloidal coomassie blue. Antigen bands were prepared and analyzed with matrix-assisted laser desorption/ionization–time of flight mass spectrometry (MALDI–TOF MS). **(B)** Examples of HIP (upper panel) and tissue-immunoprecipitation (lower panel). Labeled protein bands were analyzed in MALDI–TOF MS. Results are listed in Table 1.

Coomassie stained SDS-PAGE of eluate fractions obtained from HIP preparations generally showed lower numbers of protein bands and less IgG compared to TIP eluate fractions (Figure 3B), because washing the cryosections attached to glass coverslides removed more effectively unbound antibodies (Figure 3A). Specific antigen bands and unspecific protein bands were discriminated by comparison with eluate fractions of control sera. Using matrix-assisted laser desorption/ionization–time of flight mass spectrometry (MALDI–TOF MS), several antigens could be identified, among them: AP3B2, ATP1A3, CLIP1, CNTN1/CASPR1, CPT1C, ERC1, Flotillin1/2, GLURD2, GRIPAP1, Hexokinase-1, Homer 3, ITPR1, KCNA2, NBCe1, Neurochondrin, RGS8, ROCK2, RyR2, and STX1b (Table 1). The immunoprecipitation method which was performed for the identification of each antigen is indicated in Table 2.

**Table 1:** Proteins identified from immunoprecipitates using matrix-assisted laser desorption/ionization–time of flight mass spectrometry by peptide mass fingerprinting.

Band	Protein name/Entry name (UniProt)	Accession number	Cutoff	Score	Sequence coverage (%)	Mass^c^ (kDa)
1	AP-3 complex subunit beta-2 (**AP3B2_MOUSE**)	Q9JME5	61	78	19	119.7
2	Sodium/potassium-transporting ATPase subunit alpha-3 (**AT1A3_RAT**)	P06687	61	169	35	113.0
3	Contactin-associated protein 1 (**CNTP1_RAT**)	P97846	61	243	34	157.7
4	Contactin-associated protein 1 (**CNTP1_RAT**)	P97846	61	185	25	157.7
5	Contactin-1 (**CNTN1_RAT**)	Q63198	61	212	44	114.2
6	Glutamate receptor ionotropic, delta-2 (**GRID2_RAT**)	Q63226	61	164	36	114.0
7	Hexokinase-1 (**HXK1_RAT**)	P05708	61	154	31	103.5
8	Homer protein homolog 3 (**HOME3_RAT**)	Q9Z2X5	61	54^a^	29	39.8
9	Inositol 1,4,5-trisphosphate receptor type 1 (**ITPR1_RAT**)	P29994	61	357	34	316.4
10	Potassium voltage-gated channel subfamily A member 2 (**KCNA2_RAT**)	P63142	61	105	27	57.1
11	Electrogenic sodium bicarbonate cotransporter 1 (**S4A4_RAT**)	Q9JI66	61	69	15	122.1
12	Carnitine O-palmitoyltransferase 1, brain isoform (**CPT1C_RAT**)	F1LN46	61	211	52	91.0
13	Flotillin-2 (**FLOT2_RAT**)	Q9Z2S9	61	123	52	47.4
	Flotillin-1 (**FLOT1_RAT**)	Q9Z1E1	61	112	57	47.7
14	Syntaxin-1B (**STX1B_RAT**)	P61265	61	78	44	33.4
15	p229^b^		61	59	11	231.2
16	p75^b^		61	241	58	74.9
17	p58^b^		61	111	38	59.2
18	CAP-Gly domain-containing linker protein 1 (**CLIP1_RAT)**	Q9JK25	61	63	14	148.8
19	ELKS/Rab6-interacting/CAST family member 1 (**RB6I2_RAT**)	Q811U3	61	108	30	108.9
20	Regulator of G-protein signaling 8 (**RGS8_RAT**)	P49804	61	110	57	21.2
21	Neurochondrin (**NCDN_RAT**)	O35095	61	160	41	80.4
22	GRIP1-associated protein 1 (**GRAP1_RAT**)	Q9JHZ4	61	75	25	96.3
23	Rho-associated protein kinase 2 (**ROCK2_RAT**)	Q62868	61	225	33	161.4
24	Ryanodine receptor 2 (**RYR2_RAT**)	B0LPN4	61	376	25	567.8
25	**p48^b^**		61	188	52	47.1
26	**p51^b^**		61	119	50	50.8
27	**p43^b^**		61	87	45	43.3
28	**p41^b^**		61	95	56	40.9

*Database: SwissProt, taxonomy Mammalia*.

*All results were confirmed by mass spectrometry (MS)/MS*.

*^a^MS results not significant, antigen was confirmed by recombinant cell-based indirect immunofluorescence assay*.

*^b^Publication in progress*.

*^c^Fixed modification: carbamidomethyl (cystein); variable modification: oxidation (methionin)*.

**Table 2:** Antigens identified using histo-immunoprecipitation (HIP) or tissue-immunoprecipitation (TIP).

Protein name/Entry name (UniProt)	Gene name and/or short name	Identification method	Subcellular localization	Reactivity of control sera in RC-indirect immunofluorescence assay	%	Publication
AP-3 complex subunit beta-2 (**AP3B2_HUMAN**)	AP3B2	HIP	Cytoplasm, membrane associated	0/149 HC	0%	(19)

Sodium/potassium-transporting ATPase subunit alpha-3 (**AT1A3_RAT**)	ATP1A3	HIP	Plasma membrane	0/37 HC	0%	(20)

CAP-Gly domain-containing linker protein 1 (**CLIP1_RAT**)	CLIP1	TIP	Cytoplasm	0/49 HC^a^	0%	(21)

Contactin-1 (**CNTN1_RAT**)/Contactin-associated protein 1 (**CNTP1_RAT**)	CNTN1/CASPR1	HIP	Plasma membrane	0/48 HC 0/29 DC	0% 0%	(22)

Carnitine O-palmitoyltransferase 1, brain isoform (**CPT1C_RAT**)	CPT1C	HIP	Endoplasmic reticulum	0/44 HC	0%	

ELKS/Rab6-interacting/CAST family member 1 (**RB6I2_RAT**)	ERC1	TIP	Cytoplasm	0/49 HC 0/26 DC	0% 0%	(23)

Flotillin-1 (**FLOT1_RAT**) Flotillin-2 (**FLOT2_RAT**)	Flotillin1/2	HIP	Plasma membrane associated	0/226 HC 0/34 DC	0% 0%	(24)

Glutamate receptor ionotropic, delta-2 (**GRID2_HUMAN**)	GLURD2	HIP	Plasma membrane	0/205 HC	0%	(25)

GRIP1-associated protein 1 (**GRAP1_RAT**)	GRIPAP1	TIP	Cytoplasm, endosomes	0/50 HC^a^	0%	(26)

Hexokinase-1 (**HXK1_HUMAN**)	HK-1	HIP	Outer mitochondrial membrane	2/235 HC	0.85%	(27)
						(28)

Homer protein homolog 3 (**HOME3_RAT**)	HOMER3, Homer-3	HIP	Cytoplasm, plasma membrane associated	1/46 HC	2.17%	(29)
						(30)

Inositol 1,4,5-trisphosphate receptor type 1 (**ITPR1_RAT**)	ITPR1, IP3R1	HIP	Endoplasmic reticulum membrane	0/37 HC 0/34 DC	0% 0%	(31)

Potassium voltage-gated channel subfamily A member 2 (**KCNA2_RAT**)	KCNA2, Kv1.2	HIP	Plasma membrane	1/52 HC	1.92%	(32)

Electrogenic sodium bicarbonate cotransporter 1 (**S4A4_RAT**)	SCL4A4, NBCe1	HIP	Plasma membrane	19/235 HC 3/34 DC	8.08% 8.82%	

Neurochondrin (**NCDN_RAT**)	NCDN	TIP	Cytoplasm, partially plasma membrane associated	0/37 HC 0/33 DC	0% 0%	(33)

Regulator of G-protein signaling 8 (**RGS8_RAT**)	RGS8	TIP	Cytoplasm, plasma membrane associated	0/50 HCs^a^ 0/14 DC^a^	0% 0%	

Rho-associated protein kinase 2 (**ROCK2_RAT**)	ROCK2	TIP	Cytoplasm	0/49 HC 0/39 DC	0% 0%	(24)

Ryanodine receptor 2 (**RYR2_RAT**)	RYR2	TIP	Endoplasmic reticulum membrane	0/50 HC^a^	0%	(34)

Syntaxin-1B (**STX1B_RAT**)	STX1b	HIP	Plasma membrane associated	0/45 HC 0/33 DC	0% 0%	

p229^b^		HIP	Cytoplasm	1/148 HC	0.68%	

p75^b^		HIP	Inner mitochondrial membrane	0/48 HC 0/42 DC	0% 0%	

p58^b^		HIP	Outer mitochondrial membrane	0/44 HC	0%	

p48^b^		TIP	Cell nuclei and cytoplasm	0/48 HC 0/33 DC	0% 0%	

p41/p43/p51 complex^b^		TIP	Cytoplasm	1/49 HC^a^	2.04%	

*^a^Analyzed via ELISA with purified recombinant antigen*.

*^b^Publication in progress*.

*HC, healthy control; DC, disease control (sera with autoantibodies against known neuronal antigens)*.

### Verification

**Figure 4:**
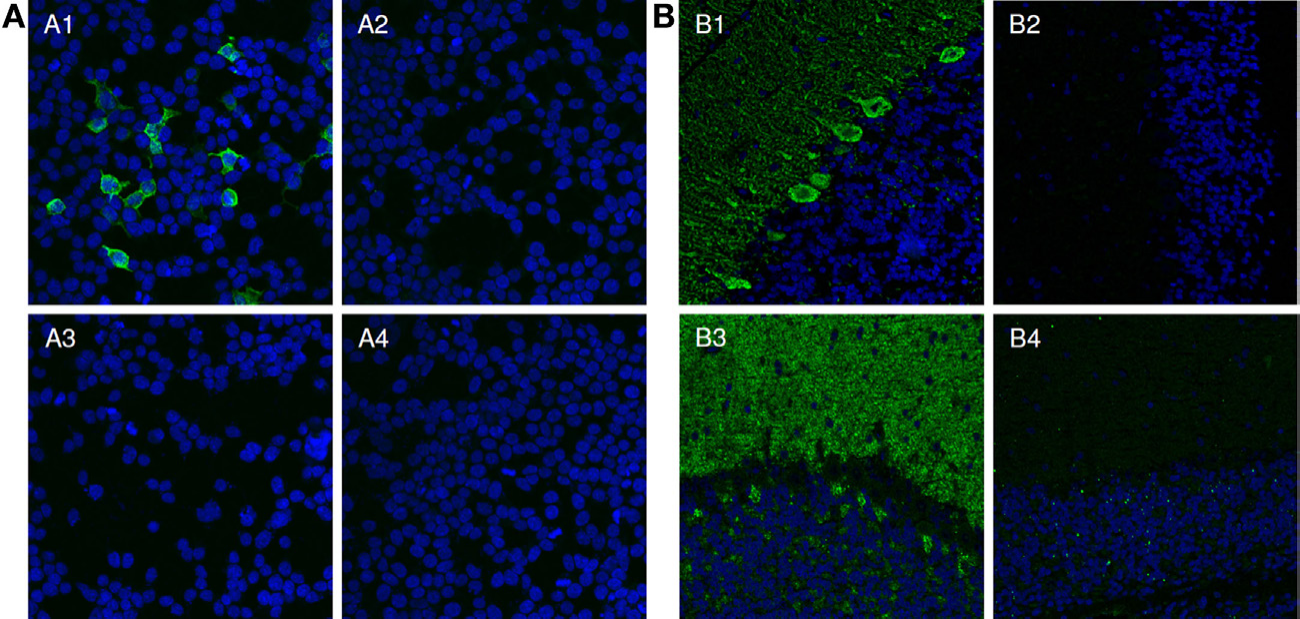
Verification of identified antigens by indirect immunofluorescence. **(A)** Indirect immunofluorescence analysis of transfected HEK293 cells. Acetone-fixed recombinant HEK293 cells expressing the protein of interest (1, 3) or a mock-transfected control (2, 4) were incubated with patient serum (1, 2) or with control serum (3, 4) (both 1:1,000). Cell nuclei were counterstained with TO-PRO-3 iodide (blue). Only HEK293-expressing the protein of interest reacted with the patient sera (green). **(B)** Two examples for the neutralization of immunofluorescence reaction on neuronal tissues. Serum 1 and 2 (green) were pre-incubated with extracts of HEK293 cells transfected with empty control vector (1, 3) or the plasmid harboring the cDNA of interest and analyzed in an indirect immunofluorescence assay with rat cerebellar cryosections (2, 4). The extract containing the protein of interest greatly reduced or abolished the immune reaction of the serum on rat cerebellum (2, 4). The control extracts had no effect (1, 3). Nuclei were counterstained by incubation with TO-PRO-3 iodide (blue).

As a proof for correct antigen identification, the patients’ samples were then tested by IFA using transfected HEK293 cells which expressed the new target antigens (Figure 4A). Characteristic staining patterns were obtained on all cellular substrates containing the respective recombinant target antigens, while there were no corresponding stainings of mock-transfected cells or cells expressing the other target antigens.

The reaction of the patients’ autoantibodies on brain tissue sections could be abolished or significantly reduced in all cases by pre-incubation with HEK293 lysate containing the respective recombinant antigen (Figure 4B). Antibody binding was unaffected when a comparable fraction from mock-transfected HEK293 cells was used.

Sera from patients with various anti-neural autoantibodies as disease controls (anti-NMDAR, anti-Hu, anti-Yo, anti-Ri, anti-AQP4, anti-LGI1, and anti-CASPR2), and sera from healthy blood donors were analyzed by IFA or ELISA with the recombinant antigens in parallel to the samples of the index patients. 18 of the 24 recombinant substrates showed no positive reactions with control sera. With five recombinant antigens only around 1–2% of the healthy blood donor sera reacted in a 1:10 dilution (Table 2).

## Discussion

Here, we describe a potent strategy to discover new neural autoantigens. Starting point is the definition of a characteristic IFA staining pattern on neural tissue. In sera of patients with putative neurological autoimmune diseases reacting with cryosections of cerebellum or hippocampus by indirect immunofluorescence but not with established brain autoantigens, we were able to identify more than 20 different target antigens. We verified all antigen identifications by (1) specific immunostaining of HEK293 cells expressing the respective recombinant antigens by the patient’s IgG and (2) specific competitive inhibition of the patient’s IgG antibody by pre-incubation with the antigen extracted from recombinant HEK293 cells.

In the first step, we analyzed the immunocomplex extractability, which determined the immunoprecipitation approach. Analyzing immunocomplex extractability rather than antigen extractability is beneficial, as autoantibody binding in first step of our HIP protocol might influence antigen solubility. The extraction buffer contained 0.5% (w/v) deoxycholate and 1% (w/v) Triton X-100 as detergents. Other solubilization agents like 0.1% SDS, as well as increased salt-concentration or pH variations could be used to adjust optimal extraction conditions for individual immunocomplexes.

In the second step, HIP was performed for extractable immunocomplexes. The antibodies bind to their targets, which are presented in their natural environment and conformation on cryosections of rat or primate cerebellum immobilized on glass slides. If HIP was not performed because of poor immunocomplex extractability, we were successful in nine cases using a classical immunoprecipitation approach by incubating tissue lysates with patients’ sera (TIP) (Table 2). In TIP, the antigens were solubilized prior to antibody binding, avoiding antigen insolubility by antibody cross linkage.

A number of techniques for the identification of autoantigens have been in use, since the discovery of autoantibodies. One approach that has been used for the discovery of neuronal surface-autoantigens in the past years relies on cultivated intact primary hippocampal neurons of rat embryos as antigen source followed by immunoprecipitation and mass spectrometry like in our protocol. Though it is focused on antibody-accessible surface proteins, and thus promises the discovery of immunosuppressable disease phenotypes it also has limitations. In particular, isolation and cultivation of these cells requires sophisticated technical skills and is highly labor-intensive. Moreover, intracellular antigens cannot be discovered by living cell-based immunoprecipitation. HIP provides better temporal flexibility as substrates can be prepared in bulk amounts and stored in aliquots for later use. In addition, cell surface and intracellular antigens are presented in their natural conformation and are accessible to antibody binding.

Compared to TIP, HIP immunoprecipitates showed fewer background bands and only weak IgG bands due to removal of unbound antibodies. This simplifies the selection of bands to be analyzed in MALDI–TOF MS and reduces false identifications. HIP also has the potential to be used for target antigen identification in patients with other organ-specific or not organ-specific autoimmune diseases.

Using HIP, five cell surface proteins could be identified (Table 2). Among them GLURD2, KCNA2, and CNTN1/CASPR1 have previously been reported as neuronal autoantigens (22, 32, 35). Out of the 19 identified intracellular antigens 7 are known antigenic targets in autoimmune diseases (19, 21, 23, 26–29, 34). Moreover, we were able to identify 14 novel autoantigens. Not all of the antigens we identified are exclusively expressed in neural tissues. For some antigens like ATP1A3, ITPR1, or ROCK2 an expression in tumor samples of the respective index patient was observed, pointing to a paraneoplastic autoimmune background (20, 31, 36). Antigens like Flotillin1/2, GRIPAP1, or CLIP1 are also expressed in non-neural tissues without a tumor association (21, 24, 26). However, other autoantibodies, like AQP4 or DPPX which are established markers for neurological autoimmune diseases (37, 38) show also expression in non-neuronal tissues.

Following the successful identification of target autoantigens based on index samples, cohort studies including patients with similar neurological symptoms and disease as well as healthy controls must validate whether the discovered autoantibodies appear rarely or are common markers for specific phenotypes like autoantibody-mediated brain disorders or paraneoplastic neurological syndromes. For autoantibodies against ATP1A3, ITPR1, NCDN, and ROCK2 an association to autoimmune cerebellar syndromes has already been demonstrated by the authors (20, 31, 33, 36). Moreover, anti-Flotillin1/2 autoantibodies were found to be present in multiple sclerosis patients (24). Thus, the antigen identification strategy that we present offers a potent instrument for identifying unknown autoantigens and contributes to better diagnosis of autoimmune diseases.

## Author Contributions

MS, SK, SH, and RM were involved in immunoprecipitation, interpretation of data, antibody testing, and microscopy. MS and RM were involved in writing of the manuscript. NB and YD performed mass spectrometric analysis. NR, CR, SB, and CP performed molecular biology work. BT and WS were involved in supervising of antibody testing and microscopy. WS and LK were involved in the conception and organization of the research project and in writing of the manuscript.

## Conflict of Interest Statement

MS, RM, SK, SH, YD, NB, NR, CR, SB, CP, BT, and LK are employees of the Euroimmun AG, a company that develops, produces, and manufactures immunoassays for the detection of disease-associated antibodies. WS is member of the Board of the Euroimmun AG.

## Abbreviations

EDTA, ethylenediamine tetraacetic acid; FITC, fluorescein isothiocyanate; HIP, histo-immunoprecipitation; IFA, indirect immunofluorescence assay; MALDI–TOF MS, matrix-assisted laser desorption/ionization–time of flight mass spectrometry; PAGE, polyacrylamide gel electrophoresis; PMF, peptide mass fingerprint; RC-IFA, recombinant cell-based indirect immunofluorescence assay; SDS, sodium dodecyl sulfate; TIP, tissue-immunoprecipitation.
