# Supplementary material for: A Spectrum of Neural Autoantigens, Newly Identified by Histo-Immunoprecipitation, Mass Spectrometry, and Recombinant Cell-Based Indirect Immunofluorescence
Source: Front Immunol. 2018 Jul 9;9:1447. doi: 10.3389/fimmu.2018.01447 (PMC6046535; doi:10.3389/fimmu.2018.01447)
Supplement: Supplementary file 1 [file table_1.docx]

| **Suppl. Table I**  Oligonucleotides and cDNAs used for cloning of the identified autoantigens | | | | | | |
| --- | --- | --- | --- | --- | --- | --- |
| **Gene** | **cDNA** | **Cleavage site** | **Oligonucleotide from 5´ to 3´ end** | **Fragment number** | **Vector** | |
| AP3B2 | IRATp970C06117D,  Source BioScience | Eco31I | ATAGGTCTCACATGTCGGCCGCCCCCGCCTACAGCG |  | pTriEx-1 | |
|  |  | Eco31I | ATAGGTCTCTTCGATCACTGGGTCAGAGCCTGTATCAC |  |  |  |
| ATP1A3 | IRAUp969H1251D,  Source BioScience | SfiI | AAGGGCCGTCAAGGCCACCATGGGGGACAAGAAAGATGACAAGGACTC |  | pTriEx-1e | |
|  |  | SfiI | TATGGCCCATGAGGCCTAGTAGTAGGTTTCCTTCTCCACCCAAC |  |  |  |
| CLIP1 | IRCMp5012B1117D. Source BioScience | BsmBI | ATACGTCTCACATGAGTATGCTAAAGCCAAGTGGGCTTAAG | Fragment 1 | pTriEx-1 | |
|  |  | BsmBI | CTGCCGTCTCTGTTCCAAGCCCTTTGCTGAAAG |  |  |  |
|  |  | BsmBI | GGCTTGGAACAGAGACGGCAGAATTTGCTG | Fragment 2 |  |  |
|  |  | BsmBI | ATACGTCTCCTCGAGGAAGGTTTCGTCGTCATTGCAGTTGG |  |  |  |
| CNTN1 | IRATp970C1261D,  Source BioScience | BsmBI | ATACGTCTCGCATGAAAATGTGGTTGCTGGTC | Fragment 1 | pTriEx-1 | |
|  |  | BsmBI | TATCGTCTCGCCTCTCACTACAAGGTCAGC |  |  |  |
|  | Gene synthesis (MWG-Biotech AG) | BsmBI | ATACGTCTCAGAGGCCCTCCCGGACC | Fragment 2 |  |  |
|  |  | BsmBI | TATCGTCTCGTCGATCAAAACTCCAGATAGACCAGAATGC |  |  |  |
| CNTNAP1 | OCABo5050E0521D,  Source BioScience | BsmBI | ATACGTCTCGCATGATGCATCTCCGGCTCTTC |  | pTriEx-1 | |
|  |  | BsmBI | TATCGTCTCGTCGATCATTCAGACCTGGACTCCTCCAGG |  |  |  |
| CPT1C | IRATp970B0237D,  Source BioScience | BsmBI | ATACGTCTCTCATGGCTGAAGCGCACCAGGCCGTGG | Fragment 1 | pTriEx-1 | |
|  |  | BsmBI | ATACGTCTCGGTCACGTACAGGAAGTCCATCATGTAATAGTTGCTGTTC |  |  |  |
|  |  | BsmBI | ATACGTCTCGTGACCCCTACACCCCTCCAGGCAGCTCGCGCTGGGAATGC | Fragment 2 |  |  |
|  |  | BsmBI | TATCGTCTCATCGAGTCAGAAGTCGGTGGATGTCATTGAGG |  |  |  |
| ERC1 | IRCBp5005F0417Q,  Source BioScience | BsmBI | ATACGTCTCTCATGTATGGAAGTGCCCGCTCTGTTGG | Fragment 1 | pTriEx-1 | |
|  |  | BsmBI | ATACGTCTCACTGCCCCCGGGTCATCTTCTGCAGCTCATTC |  |  |  |
|  |  | BsmBI | ATACGTCTCAGCAGCTTCAGGATGAGTTAGAGAAAGGTGAAC | Fragment 2 |  |  |
|  |  | BsmBI | ATACGTCTCCTCGAGTCAAGAGGACTCTTCCAGGGCGTTGAC |  |  |  |
| Flotillin 1 | IRQMp5018E075D,  Source BioScience | BsmBI | ATACGTCTCGCATGTTTTTCACTTGTGGCC |  | pTriEx-1 | |
|  |  | BsmBI | TATCGTCTCCTCGATCAGGCTGTTCTCAAAG |  |  |  |
| Flotillin 2 | Gene synthesis (MWG-Biotech AG) | BbsI | - | Fragment 1 | pTriEx-1 | |
|  |  | BbsI | - |  |  |  |
|  | IRBPp993D0417D,  Source BioScience | BbsI | ATAGAAGACTATGACGGAGAAGGAGCTCC | Fragment 2 |  |  |
|  |  | BbsI | TATGAAGACCTGCGGCGATCTTGGCAGCAATCTG |  |  |  |
|  |  | BbsI | ATAGAAGACTGCCGCACCCCTGACTAAAGTC | Fragment 3 |  |  |
|  |  | BbsI | TATGAAGACCGTCGATTACACCTGCACACCAGTGGCTTTCTTGATC |  |  |  |
| GRID2 | IRCMp5012C0927D,  Source BioScience | BbsI | ATAGAAGACGGCATGGAAGTTTTCCCCTTCCTCTTGGTTTTGTCCGTC | Fragment 1 | pTriEx-1 | |
|  |  | BbsI | ATAGAAGACAACATATCCACTGTCTTTTCAGC |  |  |  |
|  |  | BbsI | ATAGAAGACGATATGTTTGCCTGTCTTGCACC | Fragment 2 |  |  |
|  |  | BbsI | TATGAAGACGCTCGATCATATGGAGGTGCCTCG |  |  |  |
| GRIPAP1 | IRATp970G07121D,  Source BioScience | BsmBI | ATACGTCTCACATGGCGCAAGCTCTGTCTGAGGAGG |  | pET24d-N | |
|  |  | BsmBI | ATACGTCTCCTCGAGTTAGCTGGTTTCTCCTGGCTCTAGG |  |  |  |
| HK1 | IRAUp969B0528D,  Source BioScience | BveI | ATAACCTGCATGACATGATCGCCGCGCAGCTCCTGGCC |  | pTriEx-1 | |
|  |  | BveI | ATAACCTGCGATCTCGAGTTAGCTGCTTGCCTCTGTGCGTAAC |  |  |  |
| HOMER3 | IRAUp969E0465D,  Source BioScience | BsmBI | ATACGTCTCTCATGTCCACAGCCAGGGAGCAGCCAATC |  | pTriEx-1 | |
|  |  | BsmBI | ATACGTCTCTTCGAGTCAGGGCGCAGCCTCAGCCAGGCGGGC |  |  |  |
| ITPR1 | 9830108A05,  Source BioScience |  | ATAACATGTCTGACAAAATGTCGAGTTTCCTACATATTGG | Fragment 1 | |  |
|  |  |  | GCTCATTTTCACCGGAGGCTCCGTC |  |  |  |
|  | Gene synthesis (MWG-Biotech AG) |  | CAGAGGCAGGAGGTCCTTCAGGCCT | Fragment 2 | |  |
|  |  |  | CATGTTGTACAGGATCCTCTCTGCATTTTCAC |  |  |  |
|  | IRAVp968H076D,  Source BioScience |  | CATGGAAAGCAGACACGATAGTGAAAATGCAG | Fragment 3 | |  |
|  |  |  | TATGGCCCATGAGGCCTAGGCCGGCTGCTGTGGGTTGACATTC |  |  |  |
|  | Fragments 1, 2, and 3 | SfiI | GGAAGGGCCGTCAAGGCCACCATGTCTGACAAAATGTCGAGTTTCC |  | | pTriEx-1e |
|  |  | SfiI | GGTATGGCCCATGAGGCCTAGGCCGGCTGCTGTGGGTTGACATTC |  |  |  |
| KCNA2 | DNA extracted from HEK293T cells | NcoI | ATTCCATGGCAGTGGCCACCGGAGACCCAGCAGACGAG |  | | pTriEx-1 |
|  |  | XhoI | TATCTCGAGTCAGACATCAGTTAACATTTTGGTAATATTCAC |  |  |  |
| SLC4A4 | IRAMp995I1028Q,  Source BioScience | Eco31I | ATAGGTCTCACATGGAGGATGAAGCTGTCCTGGACAG | Fragment 1 | | pTriEx-1 |
|  |  | Eco31I | ATAGGTCTCTCCAGCGACTCAAGGAAAGGAATTTTATC |  |  |  |
|  | Gene synthesis (MWG-Biotech AG) | BbsI | - | Fragment 2 | |  |
|  |  | BbsI | - |  |  |  |
| NCDN | IRATp970A0738D,  Source BioScience | BbsI | ATAGAAGACAGCATGTCGTGTTGTGACCTGG |  | | pTriEx-1 |
|  |  | BbsI | ATAGAAGACTCTCGATCAGGGCTCTGACAGGCACTG |  |  |  |
| RGS8 | IRATp970H06133D,  Source BioScience | BsmBI | ATACGTCTCACATGGCGGCCTTACTGATGCCACGC |  | | pET24d-N |
|  |  | BsmBI | ATACGTCTCCTCGAGCTAACTGAGCCTCCTCTGGCTTTGG |  |  |  |
| ROCK2 | Gene synthesis (MWG-Biotech AG) | BsmBI | - | Fragment 1 | | pTriEx-1 |
|  |  | BsmBI | - |  |  |  |
|  | Gene synthesis (MWG-Biotech AG) | BsmBI | - | Fragment 2 | |  |
|  |  | BsmBI | - |  |  |  |
|  | IRCMp5012C0731D,  Source BioScience | BsmBI | ATACGTCTCTAGATATGACATACCAACTAAAAGTTATAC | Fragment 3 | |  |
|  |  | BsmBI | ATACGTCTCCTCGAGTTAGCTAGGTTTGTTTGGGGCAAGC |  |  |  |
| RYR2 | Gene synthesis (MWG-Biotech AG) | BsmBI | - |  | | pET24d-N |
|  |  | BsmBI | - |  |  |  |
| STX1B | IRAUp969B07110D,  Source BioScience | BsmBI | ATACGTCTCACATGAAGGATCGGACTCAAGAGCTGC |  | | pTriEx-1 |
|  |  | BsmBI | ATACGTCTCCTCGAGCTACAAGCCCAGCGTCCCCCCAATG |  |  |  |
