# Supplementary material for: A Spectrum of Neural Autoantigens, Newly Identified by Histo-Immunoprecipitation, Mass Spectrometry, and Recombinant Cell-Based Indirect Immunofluorescence
Source: Front Immunol. 2018 Jul 9;9:1447. doi: 10.3389/fimmu.2018.01447 (PMC6046535; doi:10.3389/fimmu.2018.01447)
Supplement: Supplementary file 2 [file image_1.PDF]

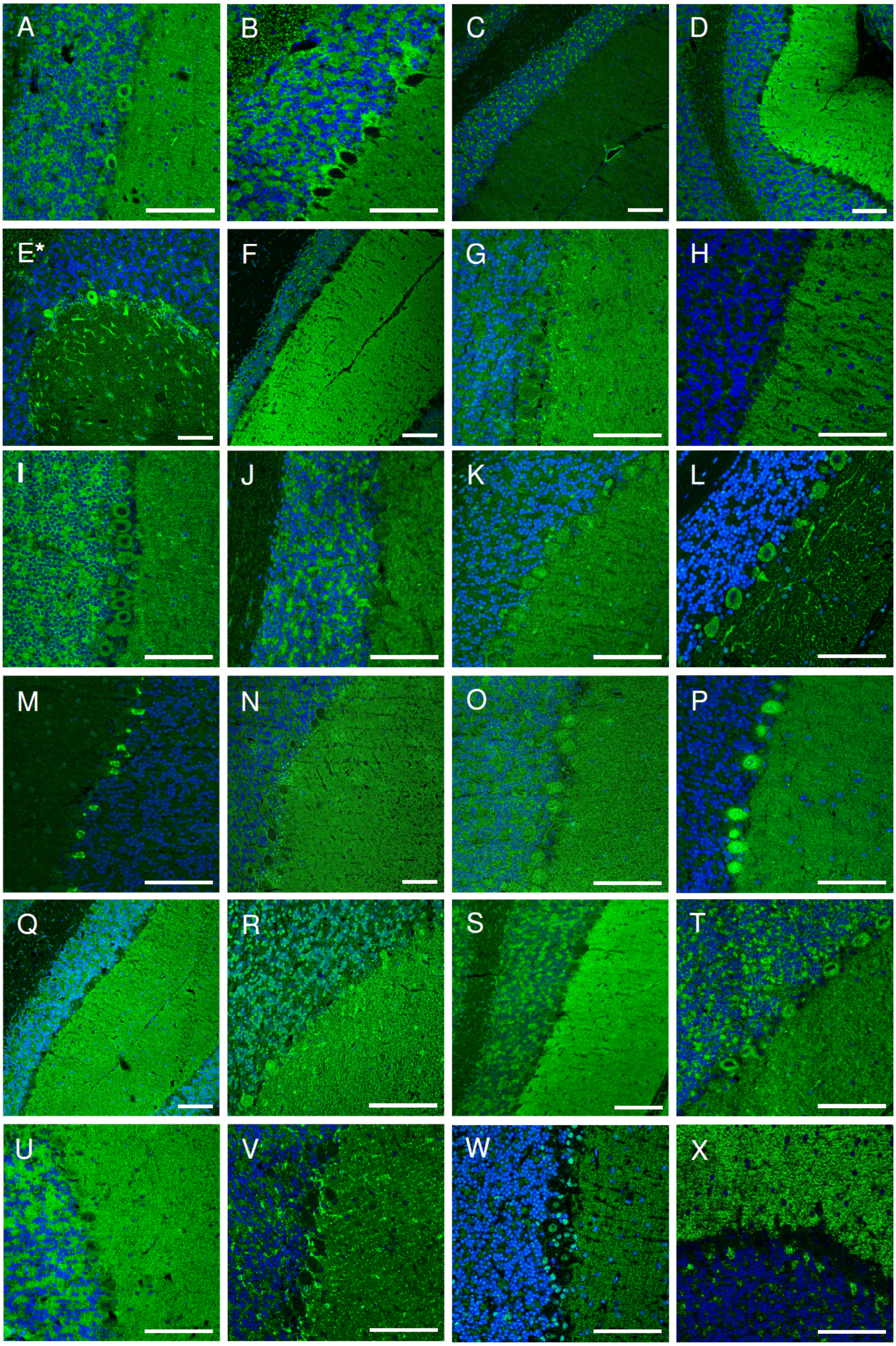
A
B
C
D
E*
F
G
H
I
J
K
L
M
N
O
P
Q
R
S
T
U
V
W
X

Suppl. Fig. 1: Immunofluorescence staining of patients' sera on cryosections of rat and monkey (*) cerebellum

Cryosections were incubated with patients' sera (**A-X**) in the first step, and with Alexa Fluor 488 labelled goat anti-human IgG in the second step (**green**). Nuclei were counterstained by incubation with TO-PRO-3 iodide (**blue**). **A:** AP3B2: granular purkinje cell cytoplasm and molecular layer and blotchy granular layer **B**: ATP1A3: smooth molecular and granular layer **C**: CLIP1: blotchy granular layer **D**: Contactin1/CASPR1: fine granular molecular layer **E:** CPT1C: granular purkinje cell cytoplasm and speckled molecular layer **F**: ERC1: granular molecular layer and blotchy granular layer **G**: Flotillin 1/2: fine granular molecular layer and blotchy granular layer, dots near the purkinje cell layer **H**: GLURD2: granular molecular layer; **I**: GRIPAP1: fine granular purkinje cell cytoplasm and molecular layer and blotchy granular layer **J:** Hexokinase 1: fine granular molecular layer and blotchy granular layer **K**: Homer3: granular molecular layer **L**: ITPR1: purkinje cell somata, axons and dendrites **M**: KCNA2: cerebellar pinceau structure **N**: NBCe1: fine granular molecular and granular layer **O**: Neurochondrin: smooth molecular layer and blotchy granular layer **P:** RGS8: smooth purkinje cell cytoplasm and fine granular molecular layer **Q:** ROCK2: granular molecular layer and blotchy granular layer **R**: RYR2: fine granular purkinje cell cytoplasm and molecular layer **S:** STX1b: smooth molecular layer and blotchy granular layer **T:** p229; **U:** p75; **V:** p58; **W:** p48; **X:** p41/p43/p51 complex. Scale bar 100 µm
